# Mannan binding lectin-associated serine protease-2 (MASP-2) critically contributes to post-ischemic brain injury independent of MASP-1

**DOI:** 10.1186/s12974-016-0684-6

**Published:** 2016-08-30

**Authors:** Franca Orsini, Elvina Chrysanthou, Thomas Dudler, W. Jason Cummings, Minoru Takahashi, Teizo Fujita, Gregory Demopulos, Maria-Grazia De Simoni, Wilhelm Schwaeble

**Affiliations:** 1 Department of Neuroscience, IRCCS-Istituto di Ricerche Farmacologiche Mario Negri, via La Masa, 19-20156, Milan, Italy; 2 Department of Infection, Immunity and Inflammation, University of Leicester, MSB, University Road, Leicester, LE1 9HN UK; 3 MRC Toxicology Unit, Leicester, LE1 9HN UK; 4 OMEROS Corporation, 201 Elliott Ave W, Seattle, WA 98119 USA; 5 Fukushima Prefectural General Hygiene Institute and Department of Immunology, Fukushima Medical University, 1 Hikariga-oka, Fukushima City, 960-1295 Japan

**Keywords:** Stroke, Cerebral ischemia, Inflammation, Complement system, Lectin pathway

## Abstract

**Background:**

Complement activation via the lectin activation pathway (LP) has been identified as the key mechanism behind post-ischemic tissue inflammation causing ischemia-reperfusion injury (IRI) which can significantly impact the clinical outcome of ischemic disease. This work defines the contributions of each of the three LP-associated enzymes—mannan-binding lectin-associated serine protease (MASP)-1, MASP-2, and MASP-3—to ischemic brain injury in experimental mouse models of stroke.

**Methods:**

Focal cerebral ischemia was induced in wild-type (WT) mice or mice deficient for defined complement components by transient middle cerebral artery occlusion (tMCAO) or three-vessel occlusion (3VO). The inhibitory MASP-2 antibody was administered systemically 7 and 3.5 days before and at reperfusion in WT mice in order to assure an effective MASP-2 inhibition throughout the study. Forty-eight hours after ischemia, neurological deficits and infarct volumes were assessed. C3 deposition and microglia/macrophage morphology were detected by immunohistochemical, immunofluorescence, and confocal analyses.

**Results:**

MASP-2-deficient mice (MASP-2^−/−^) and WT mice treated with an antibody that blocks MASP-2 activity had significantly reduced neurological deficits and histopathological damage after transient ischemia and reperfusion compared to WT or control-treated mice. Surprisingly, MASP-1/3^−/−^ mice were not protected, while mice deficient in factor B (fB^−/−^) showed reduced neurological deficits compared to WT mice. Consistent with behavioral and histological data, MASP-2^−/−^ had attenuated C3 deposition and presented with a significantly higher proportion of ramified, surveying microglia in contrast to the hypertrophic pro-inflammatory microglia/macrophage phenotype seen in the ischemic brain tissue of WT mice.

**Conclusions:**

This work demonstrates the essential role of the low-abundant MASP-2 in the mediation of cerebral ischemia-reperfusion injury and demonstrates that targeting MASP-2 by an inhibitory therapeutic antibody markedly improved the neurological and histopathological outcome after focal cerebral ischemia. These results contribute to identifying the key lectin pathway component driving brain tissue injury following cerebral ischemia and call for a revision of the presently widely accepted view that MASP-1 is an essential activator of the lectin pathway effector component MASP-2.

**Electronic supplementary material:**

The online version of this article (doi:10.1186/s12974-016-0684-6) contains supplementary material, which is available to authorized users.

## Background

Inflammatory mechanisms intrinsic to brain and blood-borne inflammatory mediators are among the major drivers of focal injury following cerebral ischemia. Among the inflammatory cascades, the complement system represents a powerful contributor to ischemic brain injury by several possible mechanisms including anaphylatoxin release, endothelial activation aiding leukocyte adhesion and recruitment, over-activation of the phagocytic system, and direct cellular lysis [1]. Activation of the complement can be achieved by three distinct pathways: the classical pathway (CP), the alternative pathway (AP), and the lectin pathway (LP). All three pathways encompass tightly regulated sequential activation cascades converging at the cleavage of the abundant complement component C3. Cleavage of C3 releases the small complement anaphylatoxin C3a and the large C3b fragment, which possess pro-inflammatory properties and promote opsonization and direct phagocytosis, respectively. In addition, C3b is also a constituent of the AP C3 convertase (C3bBb), and the deposition of C3b promotes the formation of more AP C3 convertases by binding to native factor B (fB) to form the AP zymogen complex C3bB [2]. C3b is also an essential component of both C5-cleaving convertase complexes (C3bBb(C3b)_n_ and C4b2a(C3b)_n_, respectively). With the cleavage of C5, the last enzymatic step of complement activation is completed. C5 cleavage releases the potent complement anaphylatoxin C5a and the larger fragment C5b that initiates the formation of the membrane attack complex (MAC) by subsequent recruitment of the terminal cascade components C6-C9. This MAC inserts into cell membranes to form a pore that results in ion flux, causing cell lysis.

The critical contribution of the complement system to the pathophysiology of ischemia-reperfusion injury (IRI) has been demonstrated in several models of ischemia-reperfusion injury [3–7]. It was initially hypothesized that the complement activation pathway responsible for IRI is the CP driven by natural antibodies binding to ischemia-damaged cells [8]. The involvement of the LP in IRI was first described by Collard et al. in 2000 [9], a finding that was later underlined by our own work reporting the observation that CP-deficient C1q^−/−^ mice were not protected in models of cerebral IRI [6]. The prominent role of the LP over the CP in mediating IRI was underlined by the protective phenotype of mannan-binding lectin (MBL) deficiency and the therapeutic effect of inhibitory molecules against MBL in various mouse models [7, 10–14]. Deficiency or inhibition of MBL achieved long-lasting neuroprotection and improved functional outcome in mouse models of stroke with a wide window for therapeutic intervention (up to 24 h) [13]. The latter findings are supported by numerous recent publications, which underline the prominent role of the LP in the pathogenesis and progression of brain damage in a clinical context. In stroke patients, the LP was shown to be the relevant pathway in the progression of ischemic brain damage [12, 15–17] with studies highlighting MBL [18, 19] and ficolin-3 [15], two different LP recognition molecules, as independent predictors of ischemic stroke outcome.

The initiation of the complement activation via the LP requires the binding of one or more of the five different human recognition subcomponents (i.e., MBL, collectin-11 (CL-11), ficolin-1, ficolin-2, or ficolin-3) or the five different mouse recognition components (i.e., MBL-A, MBL-C, CL-11, ficolin-A, or ficolin-B) to their cognate ligands on activating surfaces [20–22]. The ligands that mediate the binding of LP recognition components on ischemic cells are presently unknown. LP activation complexes are formed when a multimolecular complex composed of oligomers of homotrimeric recognition subunits associated with LP-specific serine proteases, called mannan-binding lectin-associated serine proteases (MASPs), bind to cognate ligands on activator surfaces. Three MASP enzymes named MASP-1, MASP-2, and MASP-3 have been described and are encoded by two different genes. The *MASP1* gene is located on human chromosome 3 (mouse chromosome 16) and encodes MASP-1 and MASP-3. The *MASP2* gene is located on human chromosome 1 (mouse chromosome 4) and encodes the serine protease MASP-2 [23, 24].

Of the three different MASPs, only MASP-2 is able to cleave both C2 and C4 to form the LP C3 convertase C4bC2a and the C5 convertase C4bC2a(C3b)_n_. Indeed, in the absence of MASP-2, but not of MASP-1/MASP-3, a complete inhibition of LP activation was observed [20, 25]. In addition, targeting MASP-2 by gene disruption or administration of antibodies that inhibit MASP-2 functional activity reduced IRI in models of myocardial, intestinal, or renal IRI [20, 26].

With respect to MASP-1, previous work suggested that, due to its ability to cleave C2 but not C4, it cannot drive LP activation in the absence of MASP-2 but may facilitate MASP-2-driven LP activation [20, 27–29]. A recent study, however, proposed that MASP-1 has an essential role in driving MASP-2 and LP activation by being an exclusive activator of MASP-2 [30], analogous to the CP serine proteases where C1r is the exclusive activator of C1s [31]. Further studies attributed an additional role to MASP-1 and/or MASP-3 in driving AP activation by converting factor D (fD) and/or fB zymogen into their enzymatically active forms [32, 33].

As for the subsequent activation steps, the activation of complement C3, but not of complement C5, was shown to be instrumental in the development of cerebral IRI, as demonstrated in a mouse model of focal ischemia [34]. Thus, it appears that C5a is not required to mediate the hallmarks of post-ischemic inflammation such as endothelial cell activation, facilitation of leukocyte adhesion and recruitment, and activation of phagocytic cells.

This study reveals the involvement of MASPs in cerebral IRI by assessing the impact of MASP-2 and of combined MASP-1 and MASP-3 deficiency in gene-targeted mouse strains and in WT mice treated with a MASP-2-specific inhibitor on functional and histopathological damage following cerebral focal ischemia. We directly compared our findings against the phenotypes seen in fB-deficient and C4-deficient mice (fB^−/−^ and C4^−/−^, respectively) analyzed in parallel.

## Methods

### Animals

Procedures involving animals and their care for transient middle cerebral artery occlusion (tMCAO) surgery were conducted at the Mario Negri Institute in conformity with institutional guidelines (Quality Management System Certificate-UNI EN ISO 9001:2008, Reg. no. 8576-A) in compliance with national (D.L. n:116,G.U. suppl. 40, February 18, 1992) and international laws and policies (EEC Council Directive 86/609, OJL 358,1; Dec. 12, 1987; NIH Guide for the Care and Use of Laboratory Animals, US National Research Council 1996, Eight Edition, 2011). All animal experiments were approved by the Mario Negri Institutional Animal Care Committee. Male 9- to 13-week-old C57Bl/6J (Charles River Laboratories, Italy) and MASP-2^−/−^ [20], MASP-1/3^−/−^ [27], C4^−/−^ [8], and fB^−/−^ [35] mice (bred at the Biomedical Services, University of Leicester) were used.

Procedures involving animals and their care for three-vessel occlusion (3VO) surgery were conducted at the University of Leicester, in accordance to the UK Animals (Scientific Procedures) Act, 1986. Female 9- to 13-week-old C57Bl/6J (Charles River Laboratories, UK) and MASP-2^−/−^ mice (Biomedical Services, University of Leicester) were used.

### MASP-2 inhibitory antibody treatment

The inhibitory MASP-2 antibody (HG4) used in this study is a derivative of the human MASP-2 inhibitory mAb OMS721 modified for improved LP inhibition in mice. HG4 and isotype control (IC) antibody (ET904, supplied by BioLegend) were administered (10 mg/kg intraperitoneally) twice (7 and 3.5 days) as single injections prior to initiation of ischemia and once (10 mg/kg intravenously), again as a single injection, at time of reperfusion.

### Transient middle cerebral artery occlusion

#### Surgery

tMCAO was induced by a siliconized filament (7-0, Doccol Corporation) introduced into the right carotid artery and advanced to block the origin of MCA for 60 min as described previously [6, 13]. Surgery-associated mortality rate was 7 %. See also Additional file 1.

#### Neurological deficits

At 48 h after tMCAO, each mouse was rated on two neurological function scales unique to mouse [5, 36]. The general deficit scale describes the well-being of the mouse, evaluating hair, ears, eyes, posture, spontaneous activity, and epileptic behavior. The focal deficit scale evaluates body symmetry, gait, climbing on a surface held at 45°, circling behavior, front limb symmetry, compulsory circling, and whisker response to a light touch. In both scales, the mice were scored from 0 (healthy mouse) to 28 (the worst performance in all categories) by a trained investigator blinded to the experimental conditions. See also Additional file 1.

#### Infarct-volume quantification

After assessment of neurological deficits, the mice were perfused and brains were obtained as described previously [6]. Twenty-micron coronal brain cryosections were cut serially and stained with cresyl violet (Sigma-Aldrich). Ischemic lesion size was calculated on seven slices by delineating the infarcted area, visualized by the relative paleness of histological staining. Infarct volumes were calculated by the integration of infarcted areas after correction for the percentage of brain swelling due to edema using Analytical Image System (Imaging Research Inc.). See also Additional file 1.

### Three-vessel occlusion

#### Surgery

3VO was conducted as described by Yanamoto et al. 2003 [37]. The two common carotid arteries (CCAs) were exposed, and the left one was clipped. The left MCA was exposed through a small burr hole in the temporal bone and permanently occluded using a bipolar coagulator. Complete ischemia was induced by clipping the right CCA for 30 min. Then, both clips on CCAs were removed, allowing reperfusion. Surgery-associated mortality rate was 8 %. See Additional file 1.

#### Infarct-volume quantification

Twenty-four hours after 3VO, the mice were sacrificed by cervical dislocation and then brains were removed, cut serially at 1-mm intervals, and stained with 2,3,5-triphenyl-2H-tetrazolium chloride (TTC) for infarct measurements using Analytical Image System. See also Additional file 1.

### Lectin pathway-specific C3 deposition assay

To assess the inhibitory effects of antibody administration on systemic LP functional activity, the sera of the mice treated with HG4 or its corresponding isotype control antibody were analyzed in LP-specific C3 deposition ELISA, as described previously [20].

### Microglia/macrophage and C3 immunofluorescence and confocal analysis

Twenty-micrometer coronal brain sections were incubated with rat anti-mouse CD11b (1:500, kindly provided by Dr. Doni, for microglia/macrophage staining) and rabbit anti-C3 polyclonal (Santa Cruz Biotechnology) primary antibodies followed by Alexa 546 anti-rat and Alexa 488 anti-rabbit (both 1:500, Invitrogen) secondary antibodies [13, 38]. Images were acquired by confocal microscopy as described previously [38]. Three-dimensional images were acquired over a 10- to 12-μm *z*-axis with a 0.23-μm step size and processed using Imaris software (Bitplane) and Photoshop CS2 (Adobe Systems Europe Ltd). See also Additional file 1.

### Microglia/macrophage and C3 immunohistochemical analysis

Immunohistochemistry was performed on 20-μm brain coronal sections using rat anti-mouse CD11b (1:800, kindly provided by Dr. Doni) and rabbit anti-C3 polyclonal (1:50, Santa Cruz Biotechnology) followed by biotinylated anti-rat and anti-rabbit secondary antibody (Vector Laboratories, CA, USA). Positive cells were stained by reaction with 3,3diaminobenzidinetetrahydrochloride (DAB, Vector laboratories). For negative control staining, the primary antibody was omitted, and no staining was observed.

For quantitative analysis of microglia/macrophage, field selection was performed on one brain coronal section (+0.1 mm from the bregma, Additional file 1: Figure S1) using a BX61 Olympus microscope equipped with a motorized stage. Frames were acquired using the software AnalySIS (Olympus) [38]. Twelve quantification fields at ×40 magnification (pixel size = 0.172 μm) were uniformly distributed over the cortex. Image processing was performed using Fiji software [39] through the algorithm previously described [40]. Once segmented, the cells were measured for the following parameters: area, Feret’s diameter (caliper), and solidity. Mean single-cell values for each parameter were used for statistics. See also Additional file 1.

For quantitative analysis of C3 staining, the entire ipsilateral cortex of one coronal section per mouse (+0.1 mm from the bregma) was acquired at ×20 by Olympus BX-61 Virtual Stage microscope, with a pixel size of 0.346 μm. Acquisition was done over 6-μm thick stacks, with a step size of 2 μm. The different focal planes were merged into a single stack by mean intensity projection to ensure consistent focus throughout the sample. The acquired area was analyzed using Fiji software. C3 staining was expressed as positive pixels/total assessed pixels and reported as the percentage of total stained area as previously described [38].

### Blinding and statistical analysis

All experimental procedures including surgery, behavioral tests, infarct-volume quantification, immunofluorescence, immunohistochemical analyses, and biochemical assays were performed by investigators blinded to the experimental conditions. Group size was defined using the following formula: *n* = 2*σ*2*f*(*α*, *β*) / Δ2 (sd in groups = *σ*, type 1 error *α* = 0.05, type II error *β* = 0.2, percentage difference between groups Δ = 20). For each measure, the standard deviation between groups was calculated on the basis of previous experiments with the same output parameters (e.g., for lesion volume quantification *σ* = 17, yielding *n* = 11.4). *p* values lower than 0.05 and 0.01 were considered significant and highly significant, respectively. Data are expressed as scatter-dot plots and means (bars). GraphPad Prism 6 software was used for statistical analysis. All the data were checked for normal distribution by Kolmogorov-Smirnov test, and unpaired *t* test or one-way ANOVA followed by Tukey’s post hoc test was used for statistical comparison among groups.

## Results

### MASP-2 significantly contributes to brain damage following cerebral IRI

**Fig. 1:**
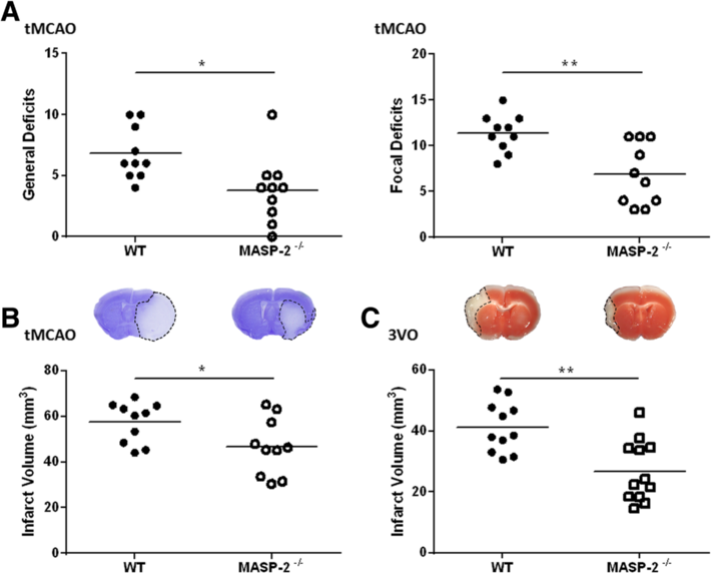
MASP-2 deficiency is protective following cerebral IRI. Neurological assessment expressed as general and focal deficits (**a**) and infarct volumes (**b**) following 60-min tMCAO and 48 h of reperfusion in WT and MASP-2^−/−^ mice, *n* = 10. Representative images of cresyl violet staining in coronal brain sections for each assessed group indicate the pale staining of the infarcted area. Infarct volume measurements following 30 min of 3VO and 24 h of reperfusion (**c**), *n* = 11–12. Representative images of TTC-stained sections indicate the infarcted area. Data are reported as scatter-dot plots and means (*bars*). *P < 0.05; **P < 0.01, unpaired *t* test. *MASP-2* mannose-binding lectin-associated serine protease 2, *WT* wild type, *tMCAO* transient middle cerebral artery occlusion, 3VO 3-vessel occlusion

First, we focused on MASP-2, the effector enzyme required for LP functional activity. Forty-eight-hours tMCAO, the MASP-2^−/−^ mice exhibited significantly lower neurological deficits when compared to the WT mice (44 and 39 % reduction for general and focal deficits, respectively, Fig. 1a). The improved neurological function in the MASP-2^−/−^ mice was associated with significantly reduced infarct volumes compared to the WT mice (19 % reduction, Fig. 1b).

Protection in the MASP-2^−/−^ mice was also observed in an additional model of stroke, the 3VO model. MASP-2 deficiency promoted tissue preservation also in this model as evidenced by a 31 % reduction in infarct volume (Fig. 1c).

**Fig. 2:**
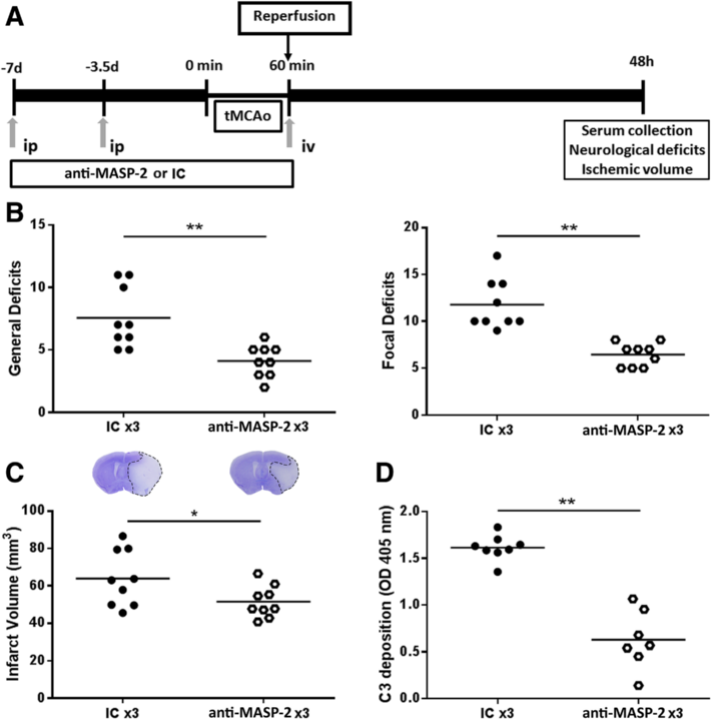
Antibody-targeted MASP-2 inhibition significantly reduces neurological deficits and infarct volumes following tMCAO. 10 mg/Kg of the anti-MASP-2 and its respective isotype control (IC) antibody were administered as single doses on 7 and 3.5 days before and 60 min after tMCAO induction (*n* = 9) (**a**). Neurological deficits (**b**) and infarct volumes (**c**) were assessed 48 h post-tMCAO. Representative images from cresyl violet staining are also shown for each of the experimental groups. Serum was collected 48 h post-tMCAO from MASP-2-antibody or IC pre-treated animals and complement functional activity tested in a lectin pathway-specific ELISA (**d**) (IC, *n* = 8; anti-MASP-2 antibody, *n* = 7). Data are presented as scatter-dot plots with means (*bars*). *P < 0.05; **P < 0.01, unpaired *t* test

To assess whether the protective phenotype observed in the MASP-2^−/−^ mice could also be obtained by pharmacological inhibition of MASP-2, the effect of MASP-2-blocking mAb was evaluated. A pharmacodynamic evaluation in naive mice indicated that single-dose intraperitoneal administration of MASP-2-blocking antibody HG4 (5 mg/kg) effectively suppressed systemic LP functional activity to undetectable levels for approximately 3 days followed by a partial pathway recovery after 7 days (Additional file 1: Figure S2). To assure effective LP inhibition throughout the study, the mice were administered HG4 or IC intraperitoneally (10 mg/kg) as a single dose 7 and 3.5 days prior to tMCAO and also received a single dose of either test agent (10 mg/kg intravenously) at time of reperfusion (Fig. 2a). Neurological assessments demonstrated that treatment with MASP-2 antibody significantly improved neurological deficits compared to equivalent dosing of IC 48 h after tMCAO (46 and 45 % reduction for general and focal deficits, respectively, Fig. 2b). Neurological improvement in MASP-2 antibody-treated mice was accompanied by significant reduction in lesion volumes (20 % reduction, Fig. 2c). A LP-specific C3 deposition assay was performed 48 h after ischemia to verify persistence of the inhibitory activity of the therapeutic antibody HG4 following injury. This assay showed that C3 deposition on mannan was significantly reduced in serum collected from HG4-treated compared to IC-treated mice (Fig. 2d) after reperfusion.

### The absence of MASP-1 and/or MASP-3 functional activity does not protect from brain damage following cerebral IRI

**Fig. 3:**
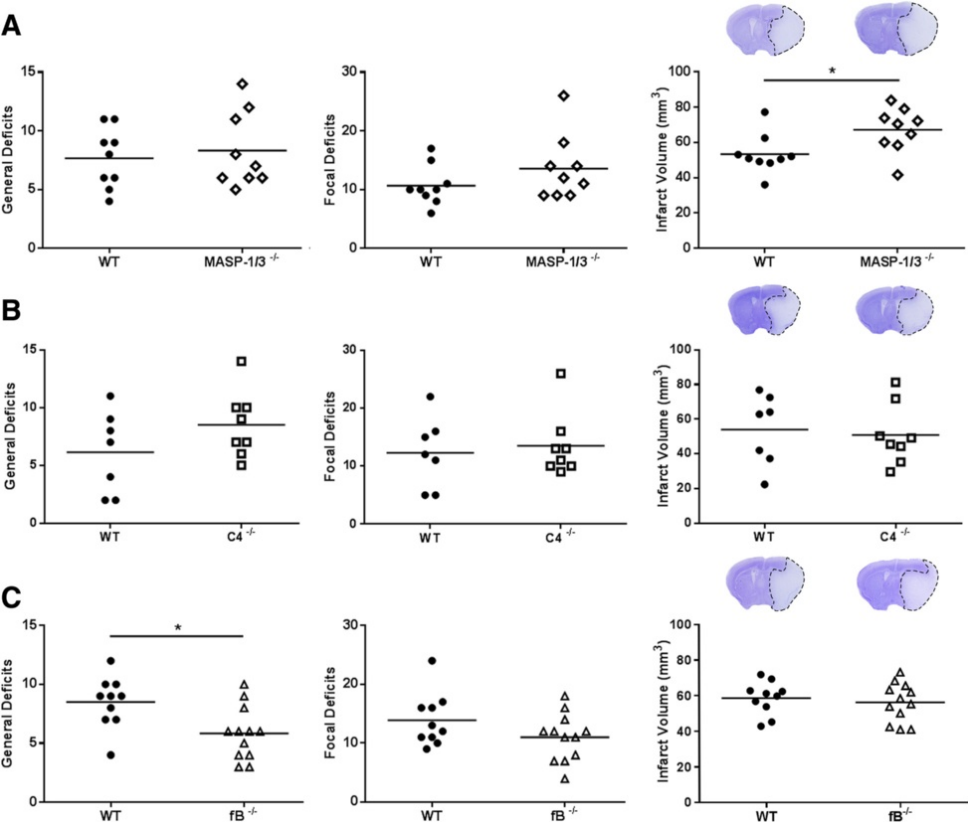
MASP-1/-3 and C4 deficiency are not protective in cerebral IRI, while fB deficiency affords a moderate degree of protection. General and focal neurological deficits and ischemic volume were assessed 48 h post-tMCAO in MASP-1/3^−/−^ (**a**), C4^−/−^ (**b**), and fB^−/−^ (**c**) mice and their WT controls (*n* = 9, *n* = 7/8, *n* = 10/12, respectively). Representative cresyl violet-stained brain slices show typical lesions. Data are reported as scatter-dot plots and mean bars. *P < 0.05, unpaired *t* test

Since it has been suggested that MASP-1 is essential for MASP-2 activation [30], we explored the post-ischemic inflammatory response in MASP-1/3^−/−^ mice. No differences in neurological deficits were observed in MASP-1/3^−/−^ when compared to WT mice, while infarct volumes were increased (Fig. 3a). These findings indicate that (i) in contrast to MASP-2, neither MASP-1 nor MASP-3 contribute to cerebral IRI following tMCAO ischemic insults and (ii) MASP-1 is not required to activate MASP-2 in an in vivo model of stroke.

### C4 deficiency does not protect from cerebral IRI

It has previously been reported that LP-mediated IRI is independent of complement C4 in models of renal and myocardial IRI. In C4-deficient human and mouse serum, a reduced but clearly detectable residual LP functional activity is maintained through a MASP-2-dependent C4-bypass activation route [20, 26]. In order to assess if this C4-bypass route is sufficient to mediate cerebral IRI, we included C4^−/−^ mice in our study. As shown in Fig. 3b, no differences in either ischemic volume or in the neurological-deficit scoring were detectable between the C4^−/−^ and WT mice 48 h after tMCAO.

### Alternative pathway functional activity is not essentially required to mediate cerebral IRI

The role of AP in cerebral IRI is still a subject of debate. We included fB^−/−^ mice, a mouse strain which completely lacks AP functional activity, for direct comparison. In these mice, the general neurological deficits were attenuated compared to the WT mice (31.8 % reduction), although focal histopathological deficits and ischemic volumes were not different from those seen in WT (Fig. 3c). These results indicate a moderate contribution of the AP in cerebral IRI as assessed 48 h after tMCAO.

### MASP-2 but not MASP-1/3 deficiency attenuates C3 deposition and reduces the pro-inflammatory microglia/macrophage phenotype in ischemic brain tissue

**Fig. 4:**
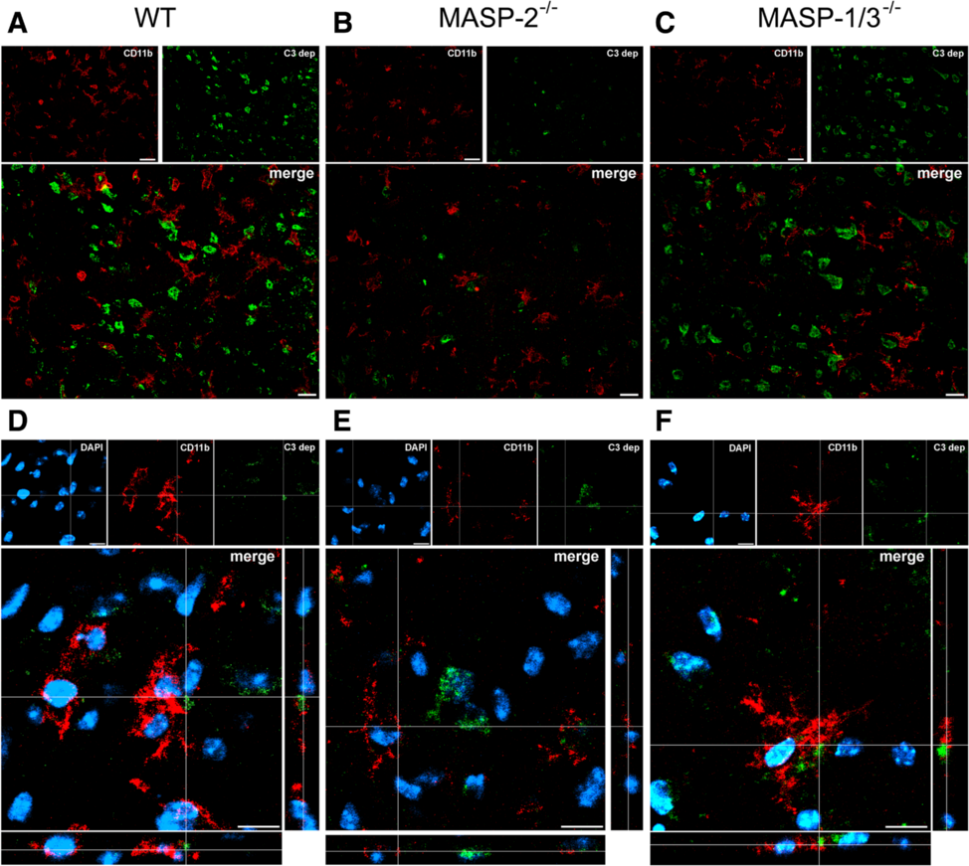
CD11b and C3 confocal co-localization in WT, MASP-2^−/−^, and MASP-1/3^−/−^ mice. Representative immunofluorescence micrographs of CD11b (*red*) and C3 deposition (*green*) in WT (**a**), MASP-2^−**/**−^ (**b**), and MASP-1/3^−**/**−^ (**c**) mice in the ischemic cortical lesion following 60 min of ischemia and 48 h of reperfusion indicating C3 reduced deposition in MASP-2^−/−^ mice (*scale bar* = 20 μm). Closer overview of CD11b-positive cells with the typical features engulfing C3-positive cells in WT (**d**) and MASP-1/3^−/−^ (**f**) and a less reactive CD11b-positive cell in close proximity to C3 in a MASP-2^−/−^ mouse (**e**). Nuclei are in *blue* (*scale bar* = 10 μm)

**Fig. 5:**
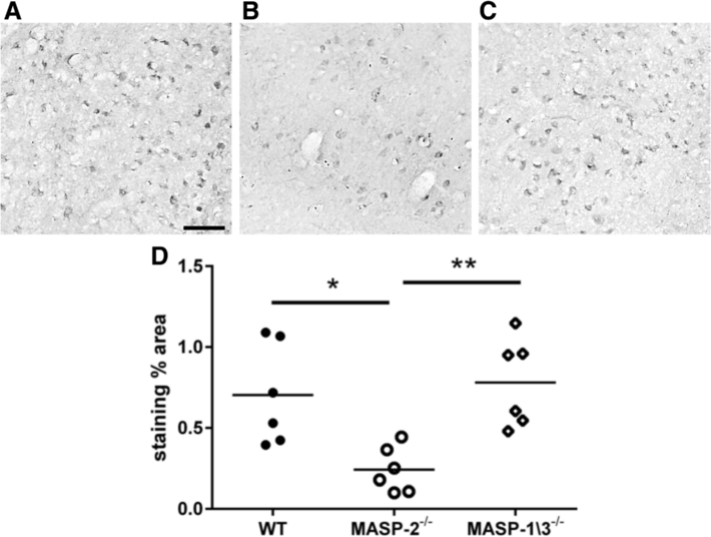
C3 immunohistochemistry in WT, MASP-2^−/−^, and MASP-1/3^−/−^ mice. Representative micrographs of C3 deposition in the ischemic cortices of WT (**a**), MASP-2^−/−^ (**b**), and MASP-1/3^−**/**−^ (**c**) mice 48 h after tMCAO (*scale bar* = 20 μm). The quantification of C3 staining shows that MASP-2^−**/**−^ mice had reduced C3 deposition when compared to WT while MASP-1/3^−/−^ were not different (**d**). Data are reported as scatter-dot plots and mean bars. *P < 0.05; **P < 0.01, one-way ANOVA followed by Tukey’s post hoc test

To further explore the effects of targeting either MASP-2 or MASP-1 and MASP-3, we analyzed C3 deposition and microglia morphology in the ischemic brain areas of the MASP-2^−/−^, MASP-1/3^−/−^, and WT mice. Immunofluorescence demonstrated that C3 deposition was markedly reduced in MASP-2^−/−^ compared to the WT or MASP-1/3^−/−^ mice (Fig. 4a–c). In addition, in the MASP-2^−/−^ mice, CD11b-positive cells, staining microglia/macrophages [38], had a decreased hypertrophic and amoeboid morphology compared to the WT or MASP-1/3^−/−^ mice (Fig. 4a–c). At higher magnification, CD11b-positive cells appeared to be hypertrophic only when in close association with C3, as detectable in the WT and MASP-1/3^−/−^ mice (Fig. 4d, f). Conversely, CD11b-positive cells had thinner ramifications with less C3 deposition seen in the MASP-2^−/−^ mice (Fig. 4e). The quantification of C3 staining on lesioned cortices confirmed that the MASP-2^−/−^ mice (Fig. 5b, d) had less C3 deposition compared to WT while MASP-1/3^−/−^ were not different (Fig. 5a, c, d).

**Fig. 6:**
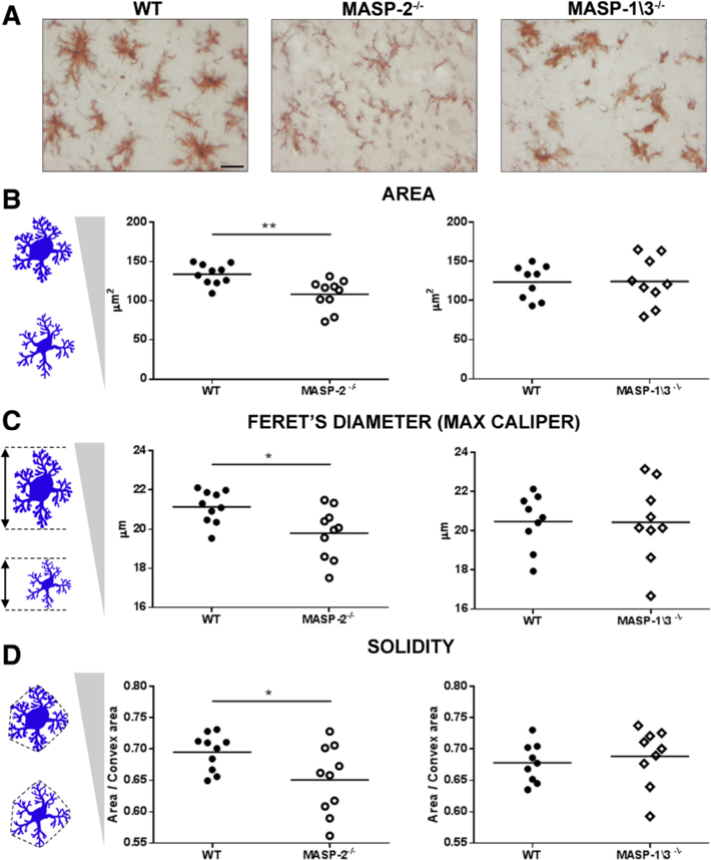
CD11b-positive cells in MASP-2^−/−^ but not in MASP-1/3^−/−^mice appear with attenuated hypertrophic and amoeboid morphology indicative of a less activated state. Representative images of CD11b-positive cells showing morphologically different cells in WT, MASP-2^−/−^, and MASP-1/3^−/−^ mice (**a**). CD11b cells in MASP-2^−/−^ mice present smaller area (**b**), lower Feret’s diameter (**c**), and lower solidity index (**d**) compared to WT. No differences were detected in MASP-1/3^−/−^ mice when compared to their WT controls. Data are reported as scatter-dot plots with means. Each *dot* represents the mean of all the cells acquired in one mouse. *P < 0.05; **P < 0.01, unpaired *t* test. *Scale bar* = 20 μm

To further define microglia/macrophage phenotypes, their morphology was characterized in the WT, MASP-2^−/−^, or MASP-1/3^−/−^ mice 48 h after ischemia (Fig. 6a). Although the percentage of total CD11b-stained areas did not differ between the WT and MASP-2^−/−^ mice (6.95 ± 1.76 and 7.13 ± 1.81 % stained area, respectively), the measurement of morphological parameters revealed that, in the MASP-2^−/−^ mice, brain microglia/macrophages predominantly maintained the shape of ramified, surveying microglia (Fig. 6a) showing significantly reduced areas (Fig. 6b), lower Feret’s diameter (Fig. 6c), and solidity values (Fig. 6d) compared to the WT mice. This strongly indicates that, in the absence of MASP-2, the prevailing microglial phenotype is that of ramified, surveying microglia while, in the WT and MASP-1/3^−/−^ mice, the predominant phenotype is that of less ramified, activated, pro-inflammatory microglia. No differences were found between the MASP-1/3^−/−^ and WT ischemic mice in CD11b-positive cell morphology parameters (Fig. 6b–d) suggesting that MASP-1/3 deficiency did not alter the state of microglia/macrophage activation.

### Brain vasculature anatomy is not affected in the different genotypes

To confirm that the ischemic injury ensues from specific complement deficiencies and not from differences in the vascular architecture among the different genotypes, we assessed the vascular features in every mouse strain used (Additional file 1: Figure S3A). The distance from the midline to the anastomotic line (defined by the anastomotic points between the anterior cerebral artery, ACA, and the middle cerebral artery, MCA) was measured at 4 and 6 mm from the frontal pole (Additional file 1: Figure S3B). The results obtained did not reveal any differences between the deficient and WT mice (Additional file 1: Figure S3C).

## Discussion

This study provides a comparative analysis of the phenotypes of mouse lines with targeted deficiencies of the LP-specific serine proteases MASP-1, MASP-2, and MASP-3 in a mouse model of stroke. Our analysis includes functional outcomes based on behavior tests (focal deficit and general neurological deficits) and histopathological outcomes (infarct sizes) as well as immunohistochemistry for deposition of the C3 activation products as a parameter for complement activation events within the ischemic areas of the brain and for morphometric endpoints (morphology of CD11b-positive macrophages/microglial cells). An AP-deficient mouse line with a targeted deficiency of the essential AP zymogen fB and a CP-deficient mouse line with a targeted deficiency in C4 were also evaluated. When compared to the WT mice, a strong protective phenotype was observed in the MASP-2-deficient mice at the level of functional and histopathological outcomes. Absence of MASP-2 lead to reduced C3 deposition in the ischemic brain area and less prominent hypertrophic and amoeboid microglial morphology. Similarly, improved outcomes were observed in the WT mice treated with a MASP-2-inhibitory antibody, corroborating the findings observed in the MASP-2^−/−^ mice.

Overall, the CD11b morphological analysis revealed that, in the absence of MASP-2 functional activity, microglial cells within the ischemic areas primarily present as ramified microglia suggestive of an anti-inflammatory polarization state [41–43] as opposed to the hypertrophic microglial morphology associated with phagocytic activity and pro-inflammatory state in the ischemic areas of WT brains. The complement system has a major role in the activation of microglia, which constitutively express receptors for C1q and for C3 cleavage products. As a consequence of complement component binding, microglia activate phagocytosis and cytokine production. Activated microglia in turn contribute to complement component production that feeds autocrine/paracrine signaling [41]. Since microglia act as a major contributor to post-injury inflammatory responses, it is plausible that complement activation through MASP-2-dependent processes could drive microglia from the ramified, surveying state towards the pro-inflammatory phagocytic phenotype, with visualized morphological changes of amoeboid cell shape, an enlarged cell soma, and retracted processes [40, 41, 44].

Since in vitro experiments suggested that MASP-1 fulfills a critical role in the activation of MASP-2 [45], we expected that the mice deficient in MASP-1 would also show protection from cerebral IRI following tMCAO. Surprisingly, the MASP-1/3^−/−^ mice presented with larger infarct volumes then their WT controls but showed a very similar degree of severity in neurological deficits. Likewise, the degree of C3 deposition within the ischemic areas in the brains of the MASP-1/3^−/−^ was similar to that seen in the WT mice. The morphometric analysis of microglia revealed no differences between the WT and MASP-1/3^−/−^ mice. The absence of any degree of protection of the MASP-1 and MASP-3 double-deficient mouse line indicates that neither MASP-1 nor MASP-3 are involved in the pathophysiological processes leading to cerebral IRI.

Several studies have indicated that MASP-1 acts as a rate-limiting protease in the activation of MASP-2 in serum. SFMI-1, a peptide that preferentially inhibits MASP-1, was reported to inhibit the LP to an extent similar to a MASP-2-specific peptide inhibitor, SFMI-2 [45]. Subsequent studies using second-generation peptide inhibitors of either MASP-1 or MASP-2 with greater specificity corroborated the initial results. These findings led to the hypothesis that, in the LP, MASP-1 is the initiating protease critically required to cleave MASP-2 zymogen into its enzymatically active form, analogous to the CP where C1r is the exclusive activator of C1s [30]. Separate in vitro studies using serum from a Malpuech–Michels–Mingarelli–Carnevale (3MC) patient lacking both MASP-1 and MASP-3 supported this hypothesis [46]. We previously reported that serum of mice deficient in both MASP-1 and MASP-3 maintains a reduced but clearly detectable LP functional activity [20, 27].

The in vivo data presented here demonstrate that targeting MASP-2 reduces cerebral IRI while the absence of MASP-1 and MASP-3 affords no protection. MASP-2 does not require MASP-1 to drive IRI. Thus, targeting MASP-1 is unlikely to disrupt the LP-mediated pathophysiological processes that lead to unfavorable outcomes.

It is undisputed that MASP-1 facilitates the conversion of zymogen MASP-2 into its enzymatically active form, most likely because the relatively low abundance of MASP-2 compared to MASP-1 is a limiting factor during the LP-specific trans-activation events involving the juxtaposition of activating complexes [47]. The non-protective phenotype of a combined MASP-1 and MASP-3 deficiency in a MASP-2-dependent pathophysiological process underlines that MASP-1 is not an essential activator of MASP-2 functional activity and highlights the fundamental differences between LP and CP activation events.

We demonstrate that targeting MASP-2 with a specific inhibitory antibody is very effective in limiting both the post-stroke neurological deficits as well as the associated ischemic lesion and in reducing the consequent activation of microglia towards the pro-inflammatory amoeboid phenotype.

This study complements our previous work in which we targeted the LP recognition subcomponents MBL-A and MBL-C by either MBL-specific mAbs or through the injection of an excess of a fluid-phase carbohydrate ligand orthologue [13]. There are advantages to targeting the single, low-abundance LP enzyme that critically drives the pathophysiology of cerebral IRI over targeting one or more of the five different LP recognition components. Mice, for example, do not express an orthologue for the human recognition subcomponent ficolin-3 (alias H-ficolin) [48]. Our own recent work has demonstrated that ficolin-3 drives LP activation in patients with subarachnoid hemorrhage [17]. In stroke patients, ficolin-1 (alias M-ficolin) serum levels are dramatically decreased at time point 6 h after the onset of symptoms, suggesting massive ficolin-1 consumption following cerebral ischemia. Ficolin-2 (alias L-ficolin) and ficolin-3 levels also decrease during the acute phase, while serum MBL levels remain unaffected [49]. These findings are in full agreement with a previously published clinical study reporting the consumption of ficolin-3 and ficolin-2 during the acute phase of stroke [15]. These reports strongly suggest that in man, ficolins are critically involved in triggering LP activation during the reperfusion phase and that blocking MBL in stroke patients might be therapeutically less effective than what we and others observed when targeting MBL in mouse models of cerebral ischemia. In addition to its low abundance, MASP-2 is exclusively synthesized in the liver [50], and the effectiveness of systemic MASP-2 inhibitory agents in target organs is not complicated by local biosynthesis.

The assessment of fB^−/−^ mice in our model of tMCAO suggests that the AP contributes to IRI following cerebral ischemia as indicated by the significant amelioration of general neurological deficits. An even stronger protection has previously been reported by Elvington et al. [51] in which a reduction in both neurological deficits and in ischemic volume were observed in fB^−/−^ mice. In our hands, the lack of AP activation does not affect the lesion size as assessed by cresyl violet staining following correction for oedema. The factor B data suggest a role for the AP in cerebral IRI by amplifying complement activation initiated by the LP. The predominant role of the MASP-2-dependent LP over the AP in the mediation of cerebral IRI pathology is underlined by the lack of a protective IRI phenotype in the MASP-1/-3 double-deficient mouse line, which is associated with low to undetectable AP functional activity [32].

The absence of a protective effect of C4 deficiency in cerebral IRI confirms the non-protective phenotype of C4 deficiency previously reported in models of myocardial and renal IRI and emphasizes the general importance of a MASP-2-dependent C4-bypass activation route [20, 22, 26].

## Conclusions

This work demonstrates the pivotal role of the LP effector enzyme MASP-2 in driving tissue injury and unfavorable outcomes in mouse models of ischemic brain ischemia and the utility of inhibitory therapeutic antibodies against this enzyme in reducing IRI-mediated tissue and organ function loss.

## Additional file

Additional file 1: Orsini_Chrysanthou. (PDF 608 kb)

## Abbreviations

CP: Classical pathway
AP: Alternative pathway
LP: Lectin pathway
MAC: Membrane attack complex
IRI: Ischemia-reperfusion injury
sCR-1: Soluble recombinant complement inhibitor
WT: Wild-type
MBL: Mannan-binding lectin
CL-11: Collectin-11
MASPs: Mannan-binding lectin-associated serine proteases
fB: Factor B
fD: Factor D
tMCAO: Transient middle cerebral artery occlusion
IC: Isotype control
3VO: Three-vessel occlusion
TTC: 2,3,5-Triphenyl-2H-tetrazolium chloride
