# Supplementary material for: Mannan binding lectin-associated serine protease-2 (MASP-2) critically contributes to post-ischemic brain injury independent of MASP-1
Source: J Neuroinflammation. 2016 Aug 30;13(1):213. doi: 10.1186/s12974-016-0684-6 (PMC5006610; doi:10.1186/s12974-016-0684-6)
Supplement: Additional file 1: — Orsini_Chrysanthou. (PDF 608 kb) [file 12974_2016_684_MOESM1_ESM.pdf]

SUPPLEMENTAL MATERIAL

SUPPLEMENTAL METHODS

Transient middle cerebral artery occlusion (tMCAO)

Surgery. Anesthesia was induced in mice by 3% isoflurane in a N_2O/O_2 (70/30%) mixture and maintained by 1-1.5% isoflurane in N_2O/O_2 (70/30%) mixture. Transient ischemia was achieved by middle cerebral artery (MCA) occlusion[1, 2] by means of a siliconized filament (7-0, Doccol Corp.) introduced into the internal right carotid artery, pushed onwards till the origin of the MCA for 60 min. At the end of the ischemic period the filament was withdrawn and reperfusion was allowed. All mice were maintained at 37°C during surgery using a heating pad (LSI Letica). Surgery-associated mortality rate was 7%.

Neurological deficits. All mice following tMCAO were assessed on an open bench top. In a neurological scoring system where 0 represents healthy and 56 the worst neurological outcome, the general and focal deficits were assessed. The general deficits describing general well being of the mice, were attributed with a maximum scoring of 28, assessing hair (0-2), ears (0-2), eyes (0-4), posture (0-4), spontaneous activity (0-4) and epileptic behaviour (0-12). Mice were assessed for focal deficits according to body symmetry (0-4), gait (0-4), climbing on a 45° surface (0-4), circling behaviour (0-4), forelimb symmetry (0-4), compulsory circling (0-4) and whisker response (0-4) with a maximum scoring of 28 [3].

Infarct volume quantification. Perfused brains were obtained as described previously[1]. Twenty-micron coronal brain cryosections were cut serially at 320 μm

intervals and stained with cresyl violet (Sigma-Aldrich). Ischemic lesion was calculated on seven slices from bregma +2 mm to -3.76 mm by delineating by the relative paleness of histological staining tracing the area on a video screen. Infarct volumes were calculated by the integration of infarcted areas after correction for the percentage of brain swelling due to edema. Edema was determined by subtracting the area of the ipsilateral from that of the contralateral hemisphere. Quantifications were performed using a computer-assisted image analyzer and calculated by Analytical Image System (Imaging Research Inc.)[3].

3 Vessel Occlusion (3VO)

Surgery. Buprenorphine (Vetergesic) diluted in saline (0.1mg/kg) was pre-operatively administered to the animals as an analgesic. Initial anaesthesia was induced with a mixture of 3 L/min isofluorane followed by maintenance anaesthesia at 1 to 2 L/min. O_2 and N_2O levels were kept constant at 1L/min and 0.8L/min respectively. Body temperature was maintained at 36°C. As described by Yanamoto *et al.* 2003 [4], via a ventral midline incision of the neck, the two common carotid arteries (CCA) were exposed followed by clamping of the left CCA using an aneurism clip. Following the left CCA clipping, the left zygomatic arch was removed to enable access to the skull and the middle cerebral artery. A 1 mm thick burr hole was opened 1 mm superior–rostral to the foramen ovale to allow access to the MCA followed by its permanent cauterisation using a bipolar coagulator (Aura, Kirwan Surgical Products). Cauterisation was performed at the lateral edge of the left olfactory tract. Following the MCA occlusion, complete ischemia was induced for 30 minutes by the clipping of the right CCA. At 30 min of ischemia, both clips were removed allowing reperfusion for 24 h. Surgery-associated mortality rate was 8%.

TTC staining. After 24h of reperfusion mice were killed via cervical dislocation and brains were removed and sliced into 1 mm thick slices using a pre-cooled brain matrix. Infarct size volume after ischemia was measured using 2, 3, 5-triphenyltetrazolium chloride (TTC). The red colouring in brain sections indicates the normal, non-infarcted tissue whereas non-coloured, white areas indicate the infarcted tissue. Brain sections were stained with 2% TTC in saline at room temperature for 30 min in the dark. Afterwards the sections were fixed in 10% formalin (Sigma) and stored in the dark at 4°C. Digital images were taken and analysed using Image J software designed to calculate the infarct volume.

Pharmacodynamic activity assay

To assess the pharmacodynamic effect of MASP-2 mAb administration in mice, systemic lectin pathway activity was evaluated by quantifying lectin-induced C3 activation in minimally diluted serum samples collected at the indicated time after MASP-2 mAb or control mAb administration to mice. Briefly, 7μm diameter polystyrene microspheres (Bang Laboratories) were coated with Mannan by overnight incubation with 33μg/mL of Mannan (Sigma) in of sodium carbonate-bicarbonate buffer (pH 9.6), then washed, blocked with 1% BSA in PBS and resuspended in PBS at a final concentration of $1x10^8$ beads/mL. Complement deposition reactions were initiated by the addition of 2.5 μL of mannan-coated beads (~250,000 beads) to 50μL of minimally diluted mouse serum sample (90% final serum concentration), followed by incubation for 40 min at 4˚C. Following termination of the deposition reaction by the addition of 250 μL of ice-cold flow cytometry buffer (FB: PBS containing 1% BSA), beads were collected by centrifugation (3000 x g for 3 min at 4˚C) and washed two more times with 300 μL of ice-cold FB.

To quantify lectin-induced C3 activation, beads were incubated for 1 hour at 4˚C with 50 µL of rabbit anti-human C3c antibody (Dako) diluted in FB. Following two washes with FB to remove unbound material, the beads were incubated for 30 min at 4˚C with 50 µL of goat anti-rabbit antibody conjugated to PE-Cy5 (Southern Biotech) diluted in FB. Following two washes with FB to remove unbound material, the beads were resuspended in FB and analyzed by a FACS Calibur cytometer. The beads were gated as a uniform population using forward and side scatter, and C3b deposition in each samples was quantified as mean fluorescent intensity (MFI).

Immunofluorescence and confocal analysis

Immunofluorescence was performed on 20-µm coronal sections as previously described[3]. Brain sections were incubated with rat anti-mouse CD11b (1:500, kindly provided by Dr.Doni) and rabbit anti-C3 polyclonal (Santa Cruz Biotechnology) primary antibodies. Alexa 546 anti-rat and Alexa 488 anti-rabbit (both 1:500, Invitrogen) were used as secondary antibodies. Appropriate negative controls without the primary antibodies were performed and none of immunofluorescence reactions revealed unspecific fluorescent signal. Immunofluorescence was acquired using a scanning sequential mode to avoid bleed-through effects by an IX81 microscope equipped with a confocal scan unitbFV500 with three laser lines: Ar-Kr (488 nm), He-Ne red (646 nm), and He-Ne green (532 nm, Olympus, Tokyo, Japan) and a UV diode. Three-dimensional images were acquired over a 10-µm z-axis with a 0.23-µm step size and processed using Imaris software (Bitplane, Zurich, Switzerland) and Photoshop CS2 (Adobe Systems Europe Ltd).

CD11b immunohistochemical analysis

Slice selection and quantitative analysis. Field selection was performed on one brain coronal section (+0.1 mm from bregma, **Fig 1S**) using a BX61 Olympus microscope equipped with a motorized stage, acquiring the focal plane corresponding to image maximal sharpness (best focus) as calculated by the acquisition software AnalySIS (Olympus)[5, 6]. Twelve quantification fields at ×40 magnification (pixel size = 0.172 µm) were uniformly distributed over the cortex. Centres of frames at ×40 were distanced 532 µm for horizontally aligned frames and 266 µm for vertically aligned frames. The gap between subsequent frames was chosen to avoid overlapping acquisitions (frame centres were distanced 89.5 and 66.5 µm from frame boundaries), thus preventing cells from appearing twice in consecutive frames.

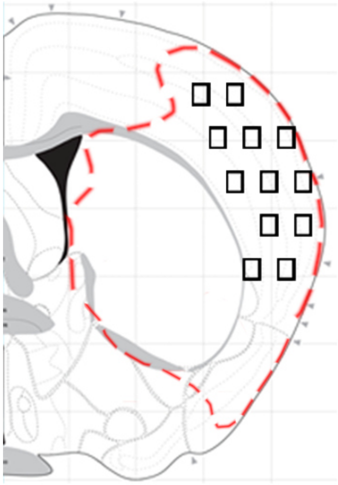

Figure 1S. Distribution of acquisition fields. In WT (A), MASP-2 KO (B) and MASP-1/3 KO mice, twelve 40x magnification fields were acquired over a cortical area involved in the ischemic lesion as evidenced by the red line. Acquisition fields were placed using a motorized stage microscope to enable unbiased sampling.

Image processing was performed using Fiji software[7] through the algorithm previously described[6]. Briefly, to ensure selecting only cells entirely present in the acquired field, cells with an area >25 µm^2 were analyzed and cells on the boundaries were excluded. Once segmented, the objects meeting the minimum size to be analyzed were measured for the following parameters: area, Feret's diameter (caliper) and solidity. Mean single cell values for each parameter were used for statistics.

Cerebral vessel staining

Cerebral vessels were stained as described by Hasan[8] with slight modifications. Mice were transcardially perfused with warm PBS followed by a mixture of two commercially available inks, Pelikan Stempelfarbe ink and Pelikan calligraphy ink (1:9, respectively). Brains were then removed and fixed overnight with 4% paraformaldehyde. The image of the right hemisphere was taken from WT and transgenic mice. The anastomotic line between the MCA and anterior carotid artery (ACA) was drawn and the distance between the brain midline and the anastomotic line was measured at 4mm and 6mm distance from the frontal pole.

SUPPLEMENTAL FIGURES

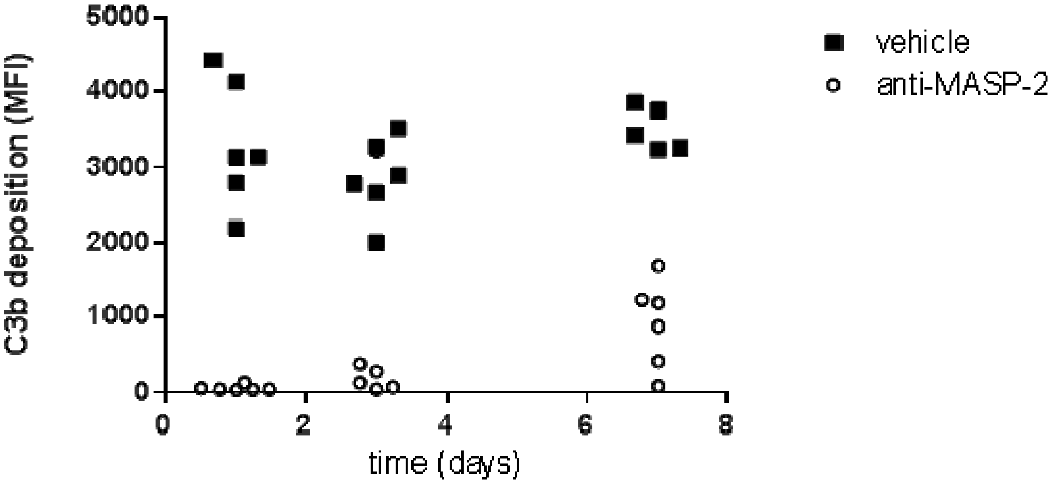

Figure 2S

Pharmacodynamics of MASP-2 mAb HG4 in mice. Groups of mice were injected intraperitoneally with 5mg/kg of mAb HG4 (open circles) or isotype control (squares). At 1 day, 3 days or 7 days after injection, mice were euthanized, bled and LP functional activity evaluated in highly concentrated serum (90%) measuring C3b deposition on mannan coated beads by flow cytometry.

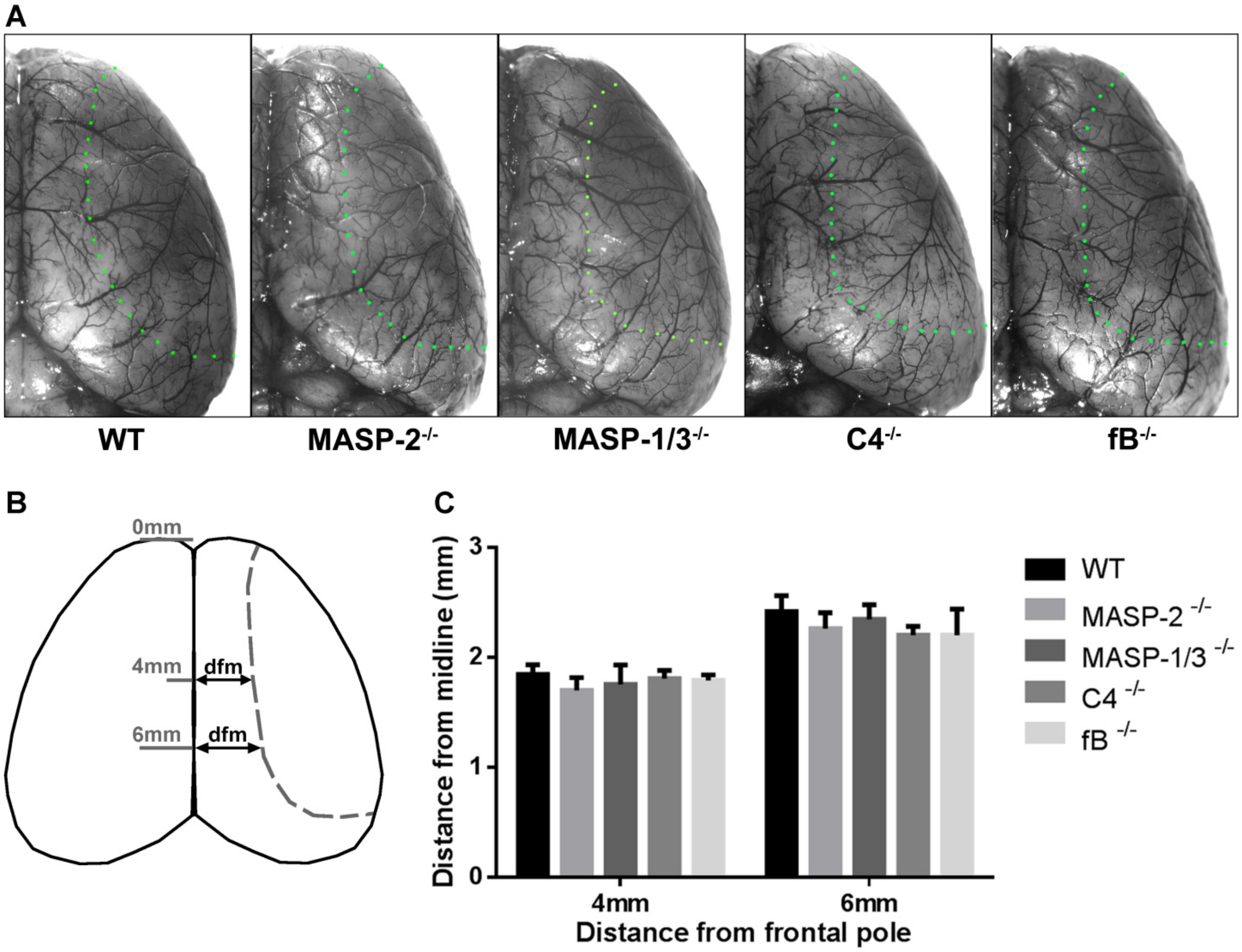

Figure 3S

There are no apparent differences in the anatomy of the cerebral vasculature amongst WT, MASP-2$^{-/-}$, MASP-1/3$^{-/-}$, C4$^{-/-}$ and fB$^{-/-}$ mice. The anastomotic line between the middle cerebral artery and the anterior cerebral artery has been drawn and shown in green in each brain of the assessed groups **(A)**. The distance of the anastomotic line to the midline at 4mm and 6mm from the frontal pole of the brain **(dfm, B)** is calculated and reported in the graph (WT and C4$^{-/-}$, n=3; MASP-2$^{-/-}$and fB, n=4; MASP-1/3$^{-/-}$, n=5)(**C**).Data are reported as column plots and SD (bars), one way ANOVA, Kruskal-Wallis test, Dunn's multiple comparisons test.

SUPPLEMENTAL REFERENCES

1. De Simoni MG, Rossi E, Storini C, Pizzimenti S, Echart C, Bergamaschini L: **The powerful neuroprotective action of C1-inhibitor on brain ischemia-reperfusion injury does not require C1q**. *Am J Pathol* 2004, **164**:1857–1863.

2. Storini C, Rossi E, Marrella V, Distaso M, Veerhuis R, Vergani C, Bergamaschini L, De Simoni M-G: **C1-inhibitor protects against brain ischemia-reperfusion injury via inhibition of cell recruitment and inflammation**. *Neurobiol Dis* 2005, **19**:10–17.

3. Orsini F, Villa P, Parrella S, Zangari R, Zanier ER, Gesuete R, Stravalaci M, Fumagalli S, Ottria R, Reina JJ, Paladini A, Micotti E, Ribeiro-Viana R, Rojo J, Pavlov VI, Stahl GL, Bernardi A, Gobbi M, De Simoni M-G: **Targeting Mannose Binding Lectin Confers Long Lasting Protection with a Surprisingly Wide Therapeutic Window in Cerebral Ischemia**. *Circulation* 2012, **126**:1484–94.

4. Yanamoto H, Nagata I, Niitsu Y, Xue JH, Zhang Z, Kikuchi H: **Evaluation of MCAO stroke models in normotensive rats: standardized neocortical infarction by the 3VO technique**. *Exp Neurol* 2003, **182**:261–274.

5. Perego C, Fumagalli S, De Simoni M-G: **Temporal pattern of expression and colocalization of microglia/macrophage phenotype markers following brain ischemic injury in mice**. *J Neuroinflammation* 2011, **8**:174.

6. Zanier ER, Fumagalli S, Perego C, Pischiutta F, De Simoni M-G: **Shape descriptors of the “never resting” microglia in three different acute brain injury models in mice**. *Intensive Care Med Exp* 2015, **3**:39.

7. Schindelin J, Arganda-Carreras I, Frise E, Kaynig V, Longair M, Pietzsch T, Preibisch S, Rueden C, Saalfeld S, Schmid B, Tinevez J-Y, White DJ, Hartenstein V, Eliceiri K, Tomancak P, Cardona A: **Fiji: an open-source platform for biological-image analysis**. *Nat Methods* 2012, **9**:676–682.

8. Hasan MR, Herz J, Hermann DM, Doeppner TR: **Intravascular perfusion of carbon black ink allows reliable visualization of cerebral vessels**. *J Vis Exp* 2013.
